# Reproducibility challenges in the search for antibacterial compounds from nature

**DOI:** 10.1371/journal.pone.0255437

**Published:** 2021-07-29

**Authors:** Nelson E. Masota, Gerd Vogg, Knut Ohlsen, Ulrike Holzgrabe

**Affiliations:** 1 Institute of Pharmacy and Food Chemistry, University of Wuerzburg, Wuerzburg, Germany; 2 School of Pharmacy, Muhimbili University of Health and Allied Sciences, Dar es Salaam, Tanzania; 3 Botanical Garden of The University of Wuerzburg, Wuerzburg, Germany; 4 Institute for Molecular Infection Biology, University of Wuerzburg, Wuerzburg, Germany; Nitte University, INDIA

## Abstract

**Background:**

Reproducibility of reported antibacterial activities of plant extracts has long remained questionable. Although plant-related factors should be well considered in serious pharmacognostic research, they are often not addressed in many research papers. Here we highlight the challenges in reproducing antibacterial activities of plant extracts.

**Methods:**

Plants with reported antibacterial activities of interest were obtained from a literature review. Antibacterial activities against *Escherichia coli* and *Klebsiella pneumoniae* were tested using extracts’ solutions in 10% DMSO and acetone. Compositions of working solutions from both solvents were established using LC-MS analysis. Moreover, the availability of details likely to affect reproducibility was evaluated in articles which reported antibacterial activities of studied plants.

**Results:**

Inhibition of bacterial growth at MIC of 256–1024 μg/mL was observed in only 15.4% of identical plant species. These values were 4–16-fold higher than those reported earlier. Further, 18.2% of related plant species had MICs of 128–256 μg/mL. Besides, 29.2% and 95.8% of the extracts were soluble to sparingly soluble in 10% DMSO and acetone, respectively. Extracts’ solutions in both solvents showed similar qualitative compositions, with differing quantities of corresponding phytochemicals. Details regarding seasons and growth state at collection were missing in 65% and 95% of evaluated articles, respectively. Likewise, solvents used to dissolve the extracts were lacking in 30% of the articles, whereas 40% of them used unidentified bacterial isolates.

**Conclusion:**

Reproducibility of previously reported activities from plants’ extracts is a multi-factorial aspect. Thus, collective approaches are necessary in addressing the highlighted challenges.

## Introduction

The discovery of novel antibiotics is urgently needed due to the ongoing challenge of antimicrobial resistance (AMR). Approaches in the search for new antibiotics include modifications of existing antibiotics, *in silico* target-based designing and synthesis of new molecules, as well as screening chemical libraries and nature. All approaches are mainly driven by the need for achieving novel antibacterial agents with novel chemical structure, target, and mode(s) of action, as well as with the absence of cross-resistance to existing antibiotics [1, 2]. Moreover, the search for compounds targeting different bacterial virulence mechanisms (pathoblockers) is a promising approach which offers lower possibilities for resistance development [3].

Nature is a potential source of hit compounds with antibacterial activity. More than half of the antibiotics currently in use are of fungi and bacterial origins. However, compounds from plants have not yet contributed to any of the antibiotics currently available on the market [4, 5]. Nevertheless, research works ranging from documentation of plants’ ethnobotanical uses to isolation and optimization of lead compounds from plants are common [4, 5]. Hence, this constitutes an important part of the search for new antibacterial compounds. These studies report on plant species, parts, nature of the extract, and bacteria species on which antibacterial activity was observed. In the case of a positive outcome, the follow-up studies typically aim at isolating, characterizing, and even optimizing the active compounds towards a lead compound, which is suitable for pre-clinical studies [6–8].

Since reporting of initial findings on antibacterial activity of plant extracts aims at providing a base for supporting further studies, a reasonably good level of reproducibility of the reported findings is crucial for preparing larger amounts of the crude extract of interest for more investigations as well as for other laboratories, who want to add other studies. Nevertheless, several factors may limit the attainment of good reproducibility level of results from Antimicrobial Susceptibility Testing (AST) of plant extracts. These include factors related to climate, soil, collection and drying practices, extraction methods as well as nature of the test bacteria [9–13]. Specifically, the determination of the anti-infective activity of extracts and the subsequently produced fractions is often performed inaccurately [13, 14], or follow old procedures, which are scientifically no longer acceptable, e.g. the use of agar diffusion assays.

This study aimed at reproducing previously reported antibacterial activities of plant extracts active against selected gram-negative bacteria from the family of Enterobacteriaceae, because we urgently need new antibiotics in this field. Further, we assessed the possibility of obtaining comparable outcomes upon the use of related plant species. Additionally, we evaluated the availability of key details regarding the plants, bacteria, and selected experimental aspects as reported in the articles from which the studied plants were obtained.

## Materials and methods

### Identification of plants with antibacterial activities

Literature search was conducted using the search string: ‘*Plant OR extract AND antibacterial OR antimicrobial OR activity AND Escherichia coli OR Klebsiella pneumoniae’*. The search was done on PubMed^®^, Web of Science^™^ and Google Scholar databases, targeting full research articles published between 1948 and 2018 in English.

Target plant species were identified by virtue of having crude extracts with moderate to high activities (Minimum Inhibitory Concentrations (MIC) of ≤256 μg/mL) against *E*. *coli* or *K*. *pneumoniae*, *as* determined by broth dilution assays [15].

### Materials

Acetone, dimethyl sulfoxide (DMSO), ethanol, ethyl acetate, n-hexane, methanol, petroleum ether, iodonitrotetrazolium chloride (INT) were purchased from Sigma-Aldrich Chemie, (Schnelldorf, Germany); Mueller Hinton Broth (MHB), Lysogeny Broth (Lennox) (LB) and agar were purchased from Carl Roth (Karlsruhe, Germany); gentamicin sulfate from AppliChem (Darmstadt, Germany) and demineralized water.

### Preparation of crude extracts

All materials were collected from fully matured plants in March, May and August 2018 from the Botanical garden of the University of Wuerzburg. Collected plant materials were kept in open paper bags and transported to the laboratory within 2 hours. The materials were then chopped into small pieces and air-dried under shade at room temperature for one to two weeks.

Dried plant materials were size reduced into coarse powders using an electric blender (Braun, Germany). Extraction was performed using 72 h maceration at room temperature and magnetic stirring. Extraction solvent types and sequences were reproduced as reported in the cited articles as indicated in Tables 2 and 3. Crude extracts solutions were obtained upon filtration and were dried under vacuum at 40 °C. Extracts obtained from solvents composed of alcohols and water (80% v/v) were further dried in a freeze dryer (Martin Christ Gefriertrocknungsanlagen, Germany) at -60 °C and 0.03–0.13 mbar. Dried extracts were weighed and stored at -15 °C until they were further used.

### Preparation of stock and working extract test solutions

Two sets of stock solutions were prepared by dissolving the extracts in 10% dimethyl sulfoxide (DMSO) and acetone, respectively, followed by ultrasonication (Bandelin electronic, Germany) for 15 minutes. Clear solutions were then obtained by centrifugation of the sonicated samples at 13000 RPMs for 10 minutes (Heraus pico 17, Thermo Fisher Scientific, Germany). 2 mL of working solutions were prepared at concentrations equivalent to 2048 μg/mL by diluting the respective volumes of stock solutions with MHB media. Following the dilutions, working solutions were mixed for 10 minutes on a lateral shaker, followed by centrifugation for 10 minutes. The concentrations of DMSO and acetone in the working solutions were 2.048 and 20.48% (v/v), respectively.

### Qualitative phytochemical screening

Qualitative phytochemical screening was done on extracts from identical plant species whose phytochemical profiles were reported in the respective publications. Qualitative tests for the presence of alkaloids, terpenes, sterols, flavonoids, phenols and tannins were carried out as per methods described by Harbone [16]. Moreover, we used LC-MS to study the profiles of an extract which phytochemical profile was established using GC-MS and LC-MS techniques. The procedure for LC-MS analysis is described in the section below.

### Solubility testing and comparative LC-MS analysis

The extents to which the extracts had dissolved in the respective solvents were semi-quantitatively evaluated based on the amount sediments at the bottom of the Eppendorf tubes after each centrifugation, as described above.

LC-MS analysis was done on selected extracts to further investigate the differences in compositions between the working solutions prepared using acetone and DMSO. Sampling of the extracts was done to include extracts with better solubility in acetone (*C*. *longa* and *G*. *tinctoria*) as well as in 10% DMSO (*F*. *carica*) (Table 4).

In that, stock solutions equivalent to 10 mg/mL were prepared from the extracts in acetone and 10% DMSO, followed by 10 min. ultrasonication and centrifugation at 13000 RPMS for 10 min. To prepare working solutions, 1 mL of the stock solutions diluted with 4 mL of each distilled water, with subsequent 10 min ultrasonication and centrifugation at 13000 RPMS for 10 min.

To remove the solvents, 2 mL of the supernatant from each working solution were transferred to 10 mL preparation glasses and were freeze dried for 24 h. Solution for LC-MS analysis were thereafter obtained by reconstituting the dried residues with 1 mL of methanol. Analysis was conducted by injecting 10 μL of each obtained solution into an LCMS-2020 (Shimadzu, Japan) system, using previously reported chromatographic conditions [17].

### Antimicrobial susceptibility testing

Since a broad range of bacterial strains were used in the referred studies, we opted to use reference *Escherichia coli* (ATCC 25922) and *Klebsiella pneumoniae* (ATCC 10031) strains. The two strains have no resistance mechanisms to antibiotics and were obtained from the American Type Culture Collection (ATCC).

Overnight bacteria cultures were prepared by suspending one colony from LB agar plates into 2 mL of autoclave sterilized LB broth followed by 24 h incubation at 37 °C under lateral shaking (Edmund Bühler GmbH, Germany). Fresh cultures were then prepared by transferring 200 μL of the overnight culture into 20 mL of LB broth with a subsequent incubation for 6 h at 37 °C under lateral shaking. The number of Colony Forming Units (CFU) in the fresh cultures was determined using the optical densities (Eppendorf BioPhotometer Plus, Eppendorf AG, Germany) of ten-times diluted fresh cultures and Newman’s correlation curve. Bacterial suspensions containing 10^6^ CFU/mL of each bacteria were prepared by drawing the appropriate volume of the fresh culture into the corresponding amount of MHB media. These suspensions were used within 30 minutes from their preparation [18, 19].

Antimicrobial susceptibility testing was done using broth microdilution assays on 96 wells microtiter plates (Greiner Bio-One, Austria). Using a multichannel pipette (Eppendorf AG, Germany), 100 μL of autoclave MHB media was added in triplicates into wells in rows 2 to 12, followed by 200 μL of the extract test solution in the first row. A serial two-fold dilution was then done by drawing 100 μL of the extract solution from the first row and mixing with 100 μL of MHB media in the second row. The procedure was repeated to the last row, whereby the final 100 μL was discarded [18, 19].

Following the serial dilution, 100 μL of 10^6^ CFU/mL bacteria suspension in MHB media were added into the respective wells on the microtiter plate in triplicates. Each extract was tested in a range of 0.5–1024 μg/mL. The highest concentration of DMSO and acetone, respectively in the first wells were 1.024 and 10.24%(v/v). Gentamicin sulfate was used as a positive control in a range of 0.0156–32 μg/mL. Negative control involved solutions of DMSO and acetone, respectively, in MHB media at 1.28%(v/v) and 10.24%(v/v). Other controls in place included sterility controls for crude extracts and MHB media, as well as bacteria growth controls. All extracts and controls were tested in triplicates and the experiments were repeated twice in accordance to references [13, 20, 21].

The loaded plates were incubated for 18 h at 37 °C (Hera Cell incubator, Heraeus, Germany); then 40 μL of 0.2 mg/mL solution of iodonitrotetrazolium chloride (INT) was added into all wells and further incubated for 30 minutes. The MICs were determined by visual observation for wells with no formation of pinkish coloration, indicating the absence of actively diving bacteria.

## Results

From the literature search, 204 plant species were identified to meet our set criteria of having either crude extracts or volatile/essential oils with MICs ≤256 μg/mL against *E*. *coli* or *K*. *pneumoniae*, determined by broth dilution assays. Upon excluding studies, which tested volatile or essential oils, 40 species were noted to be available in the Botanical garden of the University of Wuerzburg as either identical or related species. Moreover, due to the limited availability of parts like nuts, fruits and pericarps, matching plant parts were obtained from only 13 identical and 11 related species (Tables 1–3). The growing conditions for the plants corresponded to their natural habitats with regard to temperature and relative humidity (Table 1).

**Table 1:** Plants studied.

SN	Name (Family)	Plant part	Internal accession number	Growth area
1	*Castanea sativa* Mill. (Fagaceae)	Leaves	XXXX-539-H-60	outdoor
2	*Cinnamomum verum* L. (Lauraceae)	Leaves	2010-90-B-80	Glasshouse—tropical^a^
3	*Datura stramonium* L. (Solanaceae)	Seeds	XXXX-868-G-74	outdoor
4	*Juniperus oxycedrus* L. (Cupressaceae)	Leaves	XXXX-925-G-80	outdoor
5	*Murraya koenigii* (L.) Spreng. (Rutaceae)	Leaves	2004-70-B-80	Glasshouse—tropical^a^
6	*Olea europaea* L. (Oleaceae)	Leaves	XXXX-954-G-80	Glasshouse—Mediterranean^b^
7	*Piper betle* L. (Piperaceae)	Leaves	1990-349-D-80	Glasshouse—tropical^a^
8	*Ricinus communis* L. (Euphorbiaceae)	Seeds	XXXX-1004-G-74	outdoor
9	*Salvia officinalis* L. (Lamiaceae)	Leaves	XXXX-1015-G-80	outdoor
10	*Satureja hortensis* L. (Lamiaceae)	Aerial parts	XXXX-596-G-70	outdoor
11	*Silybum marianum* (L.) Gaertn. (Asteraceae)	Seeds	XXXX-1080-G-74	outdoor
12	*Viscum album* L. (Loranthaceae)	Leaves	XXXX-1072-H-70	outdoor
13	*Zingiber officinale* Rosc. (Zingiberaceae)	Rhizomes	Charge 329272 (Kraeuter Mix, Germany)	outdoor
14	*Acacia melanoxylon* R.Br. (Fabaceae)	Leaves	XXXX-399-E-80	Glasshouse—cultivation area^c^
15	*Acacia retinoides* Schltdl. (Fabaceae)	Leaves	XXXX-106-P-70	Glasshouse—cultivation area^c^
16	*Adiantum raddianum* C.Presl. (Adiantaceae)	Whole plant	2001-62-B-80	Glasshouse—cultivation area^c^
17	*Alpinia purpurata* (Vieill.) K. Schum. (Zingiberaceae)	Leaves	2010-88-B-70	Glasshouse—tropical^a^
18	*Curcuma longa* L. (Zingiberaceae)	Rhizome	2004-25-D-80	Glasshouse—tropical^a^
19	*Erythrina crista-galli* L. (Fabaceae)	Bark	1982-348-E-80	Glasshouse—Mediterranean^b^
20	*Ficus carica* L. (Moraceae)	Bark	XXXX-220-G-80	outdoor
21	*Garcinia spicata* Hook.f. (Clusiaceae)	Leaves	1977-306-D-80	Glasshouse—tropical^a^
22	*Garcinia tinctoria* (DC.) W. Wight (Clusiaceae)	Leaves	XXXX-74-B-80	Glasshouse—tropical^a^
23	*Paeonia officinalis* L. (Paeoniaceae)	Leaves	2013-11-S-10	outdoor
24	*Satureja montana* L. (Lamiaceae)	Aerial parts	XXXX-1068-K-80	outdoor

^**a**^ temperature = 18–25 °C, relative humidity = 70–90%;

^**b**^ temperature = 4–25 °C, relative humidity = 50–70%;

^**c**^ temperature = 8–25 °C, relative humidity = 50–70%.

**Table 2:** Minimum inhibitory concentrations (MICs) of 10% DMSO and acetone dissolved extracts of plant species identical to those reported in the literature.

Studied plants and extractants			Minimum inhibitory concentration (μg/mL)						
			10% DMSO dissolved extract		acetone dissolved extracts		Previously reported antibacterial activities		
Sn	Plant’s name	Extracting solvent	Ec (ATCC 25922)	Kp (ATCC 10031)	Ec (ATCC 25922)	Kp (ATCC 10031)	Ec	Kp	Ref.
1	*Castanea sativa* Mill.	ethyl acetate	>1024	>1024	>1024	>1024	256	256	[25]
2	*Cinnamomum verum* L.	methanol	>1024	**1024**	>1024	**1024**	64	256	[23]
3	*Datura stramonium* L.	petroleum ether	>512	>512	>512	>512	39.1	-	[26]
4	*Juniperus oxycedres* L.	methanol	>1024	>1024	>1024	>1024	250	-	[27]
5	*Murraya koenigii* (L.) Spreng.	acetone (benzene*)	>1024	>1024	>1024	>1024	125	250	[28]
6	*Olea europaea* L.	acetone	>1024	>1024	>1024	>1024	60	25	[24]
7	*Piper betle* L.	ethanol	>1024	>1024	>1024	**1024**	250	250	[29]
8	*Ricinus communis* L.	methanol	>1024	>1024	>1024	>1024	250	31	[28]
9	*Salvia officinalis* L.	acetone	>1024	>1024	>1024	>1024	-	156	[30]
10	*Satureja hortensis* L.	methanol	>1024	>1024	>1024	>1024	250	-	[31]
11	*Silybum marianum* (L.) Gaertn.	ethanol-water 8:2 v/v	>1024	>1024	>1024	>1024	41.2	20	[32]
12	*Viscum album* L.	methanol	>1024	>1024	>1024	>1024	256	256	[22]
13	*Zingiber officinale* Rosc.	ethanol	>512	>512	>512	>512	75.6	185.5	[33]

* Solvent used in the reference article but avoided in this study due to toxicity concerns

**Table 3:** Minimum inhibitory concentrations (MICs) of 10% DMSO and acetone dissolved extracts of plant species reported to those reported in the literature.

Studied plants and extractants				Minimum inhibitory concentration (μg/mL)						
				DMSO dissolved extract		acetone dissolved extract		Previously reported antibacterial activities		
Sn	Studied species	Species reported in literature	Extracting solvent	Ec (ATCC 25922)	Kp (ATCC 10031)	Ec (ATCC 25922)	Kp (ATCC 10031)	Ec	Kp	Reference
1	*Acacia melanoxylon* R.Br.	*Acacia nilotica*	ethanol 80%	>1024	>1024	>1024	>1024	19.5	9.75	[34]
2	*Acacia retinoides* Schltdl.	*Acacia nilotica*	ethanol 80%	>1024	>1024	>1024	>1024	19.5	9.75	[34]
3	*Adiantum raddianum* C.Presl.	*Adiantum venustum*	methanol	>1024	>1024	>1024	>1024	15.6	7.81	[35]
4	*Alpinia purpurata* (Vieill.) K. Schum.	*Alpinia galanga* (L.) Wild	methanol	>1024	>1024	>1024	>1024	80	160	[36]
5	*Curcuma longa* L.	*Curcuma malabarica*	n-hexane	>1024	>1024	>1024	**1024**	-	10	[37]
6	*Erythrina crista-galli* L.	*Erythrina sigmoidea*	methanol	>1024	>1024	>1024	>1024	16	64	[38]
7	*Ficus carica* L.	*Ficus bubu* Warb.	methanol	>1024	>1024	>1024	>1024	39.1	-	[39]
8	*Garcinia spicata* Hook.f.	*Garcinia smeathmannii* Oliver	methanol	>1024	**512**	>1024	**512**	39.1	78.1	[40]
9	*Garcinia tinctoria* (DC.) W. Wight	*Garcinia smeathmannii* Oliver	methanol	>1024	>1024	>1024	>1024	39.1	78.1	[40]
10	*Paeonia officinalis* L.	*Paeonia broteroi* Boiss. & Reut.	acetone (Multiple*)	**256**	**128**	**256**	**128**	-	250	[41]
11	*Satureja montana* L.	*Satureja hortensis* L.	methanol	>1024	>1024	>1024	>1024	250	-	[31]

* Five extraction solvents of varying polarities were used in the reference article, all giving the same MIC value.

Plants’ details such as taxonomy, location and season of collection, growth state at collection, and parts studied were obtained from the reviewed articles as far as reported. Furthermore, we gathered information on solvents used for extraction, type of the bacterial strains studied, reference AST methods used, bacteria growth visualizing techniques as well as positive and negative controls applied.

We observed a very low reproducibility of antibacterial activities in extracts from plant species identical to those previously reported (Table 2). Only extracts from *Cinnamomum verum* L. and *Piper betle* L. showed MICs within the tested concentration (1024 μg/mL) against at least one of the bacteria tested.

Among the related species, extracts from *Garcinia spicata* Hook. f. showed MICs of 512 μg/mL against *K*. *pneumoniae*, whereas *Paeonia officinalis* L. inhibited the growth of both *E*. *coli* and *K*. *pneumoniae* at MICs of 256 μg/mL and 128 μg/mL, respectively (Table 3). All MICs are rather high.

Generally, the screening for phytochemicals present in the extracts studied was observed to be uncommon in literature. In our study, we could obtain results on qualitative phytochemical screening in only 3 out of 13 compared identical plant species, whereas no quantitative screening was reported This hindered a large qualitative and quantitative comparison in this aspect.

However, the methanol leaf extract of *V*. *album* was reported to contain alkaloids, terpenes, sterols, flavonoids and polyphenols, whereas a similar extract in our study did not contain sterols [22]. Moreover, our results were similar to those reported in the methanol leave extract of *C*. *verum*, which contained alkaloids, flavonoids, phenols, sterols and tannins [23]. The observed antibacterial activities in *V*. *album* and *C*. *verum* were not directly associated to a specific type of phytochemicals found to be present in the extracts. Korukluoglu *et al*. used GC-MS and LC-MS to screen for compounds present in an acetone extract of *O*. *europaea* leaves. Similar to their findings, our LC-MS analysis of the corresponding extract showed the presence of vanillic acid (*m/z* 312), syringic acid (*m/z* 342), *p*-coumaric acid (*m/z* 308), ferulic acid (*m/z* 338), and oleuropein (*m/z* 540). Moreover, the presence of three other compounds each with a mass to charge ratio 282 (4-hydroxybenzoic acid, veratric acid and protocatecuic acid) could not be verified with certainty since we observed only one peak corresponding to *m/z* 282, whereas caffeic acid (*m/z* 396) was not present. However, the referred study reported inhibitory effect of caffeic acid against *E*. *coli* and *K*. *pneumoniae*, among other bacteria [24].

Upon preparing stock and working solutions, most crude extracts dissolved to a greater extent in acetone as compared to 10% DMSO. This observation, however, did not result in notable differences in the observed antibacterial activities of the solutions (Table 4).

**Table 4:** Semi-quantitative evaluation of solubility of crude extracts in 10% DMSO and acetone.

Sn	Plant’s name	Part	Extracting solvent	Dissolving extent of crude extract in:			
				10% DMSO		acetone	
				stock	working	stock	working
1	*Castanea sativa* Mill.	Leaves	ethyl acetate	+	++	++	+
2	*Cinnamomum verum* L.	Leaves	methanol	+	++	++	++
3	*Datura stramonium* L.	Seeds	petroleum ether	+	+++	+++	+++
4	*Juniperus oxycedres* L.	Leaves	methanol	++	++	++	++
5	*Murraya koenigii* (L.) Spreng.	Leaves	acetone	+	+++	++	++
6	*Olea europaea* L.	Leaves	acetone	+	+++	+++	+
7	*Piper betle* L.	Leaves	ethanol	++	++	++	++
8	*Ricinus communis* L.	Seeds	methanol	+	+++	++	+++
9	*Salvia officinalis* L.	Leaves	acetone	+	+++	++	+
10	*Satureja hortensis* L.	Aerial parts	methanol	+	++	++	++
11	*Silybum Marianum* (L.) Gaertn.	Seeds	ethanol 80%	+	+++	++	+++
12	*Viscum album* L.	Leaves	methanol	+	++	++	+
13	*Zingiber officinale* Rosc.	Rhizomes	methanol, ethanol	+	+++	+++	++
14	*Acacia melanoxylon* R.Br.	Leaves	ethanol 80%	+++	++	++	++
15	*Acacia retinoides* Schltdl.	Leaves	ethanol 80%	+	+++	++	++
16	*Adiantum raddianum* C.Presl.	Whole plant	methanol	++	++	++	++
17	*Alpinia purpurata* (Vieill.) K. Schum.	Leaves	methanol	+++	++	++	++
18	*Curcuma longa* L.	rhizome	n-hexane	+	++	+++	+++
19	*Erythrina crista-galli* L.	Bark	methanol	+	+++	++	++
20	*Ficus carica* L.	Bark	methanol	++	+++	+	++
21	*Garcinia spicata* Hook.f.	Leaves	methanol	++	++	++	+++
22	*Garcinia tinctoria* (DC.) W. Wight	Leaves	methanol	+	++	++	+++
23	*Paeonia officinalis* L.	Leaves	acetone	+	++	+++	++
24	*Satureja montana* L.	Aerial parts	methanol	+	++	++	+

+++ = Soluble; ++ = sparingly soluble; + = slightly soluble

**Fig 1:**
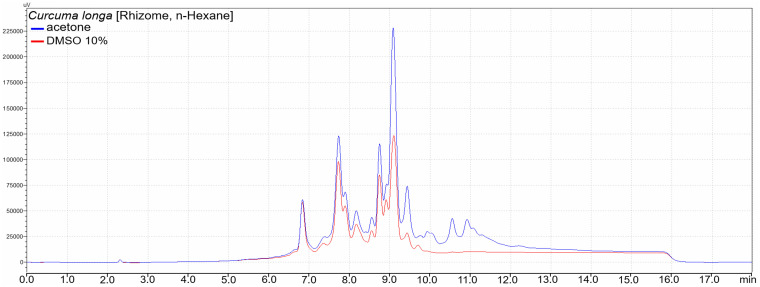
An overlay of UV chromatograms of acetone (blue) and DMSO (red) based working solutions of an n-hexane rhizomes extract of *C*. *longa*. Qualitative similarity and higher quantities of phytochemicals observed in the acetone based working solution, which had better solubility compared to that of DMSO.

LC-MS analysis of working solutions prepared using acetone and 10% DMSO showed that the extracts were qualitatively similar to one another. However, the solutions had some quantitative differences. For example, higher quantities of phytochemical were observed in n-hexane rhizome extract of *C*. *longa* dissolved in acetone compared to 10% DMSO (Fig 1), because the extract showed better solubility in acetone (Table 4). In addition, additional peaks were noted in the low polarity region of the acetone dissolved extract’s chromatogram (Fig 1).

**Fig 2:**
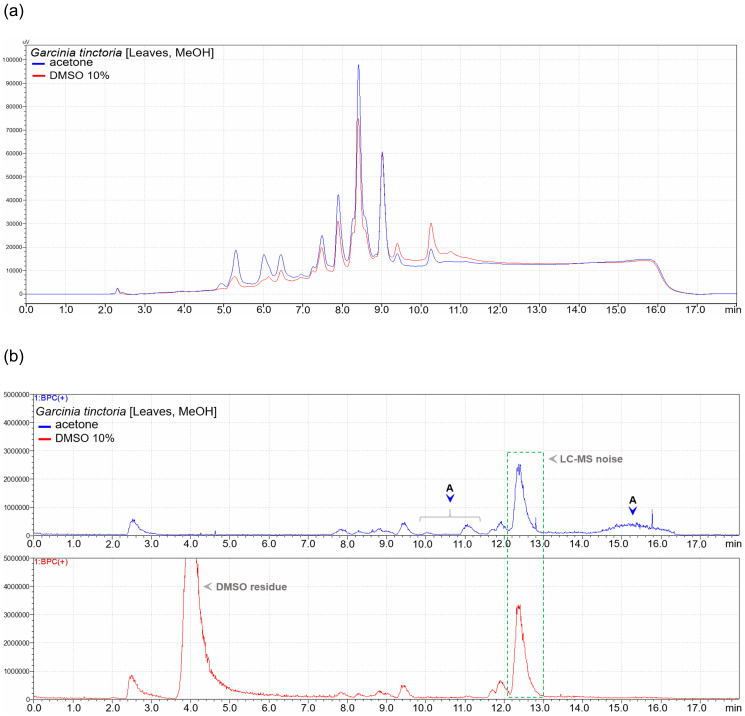
**a**. An overlay of UV chromatograms of acetone (blue) and DMSO (red) based working solutions of a methanol leaves extract of *G*. *tinctoria*, which had better solubility in acetone. Higher quantities of phytochemicals are observed in the acetone based working solution towards a less polar region of the chromatogram. **b**. Comparison of base peak chromatograms of acetone (blue) and DMSO (red) based working solutions of a methanol leaves extract of *G*. *tinctoria*, which had a better solubility in acetone. At least three additional compounds (marked A) are visible in the less polar region of acetone based working solution’s chromatogram.

Moreover, when dissolved in acetone, *G*. *tinctoria* leaves extract from a more polar extracting solvent (methanol) showed higher quantities of phytochemicals in the polar region of the chromatogram, as compared to the less polar region (Fig 2a) (Table 4). The Base Peak Chromatograms (BPC) of the same solutions indicated the presence of at least 3 additional compounds in the less polar region of the acetone based working solution (Fig 2b).

**Fig 3:**
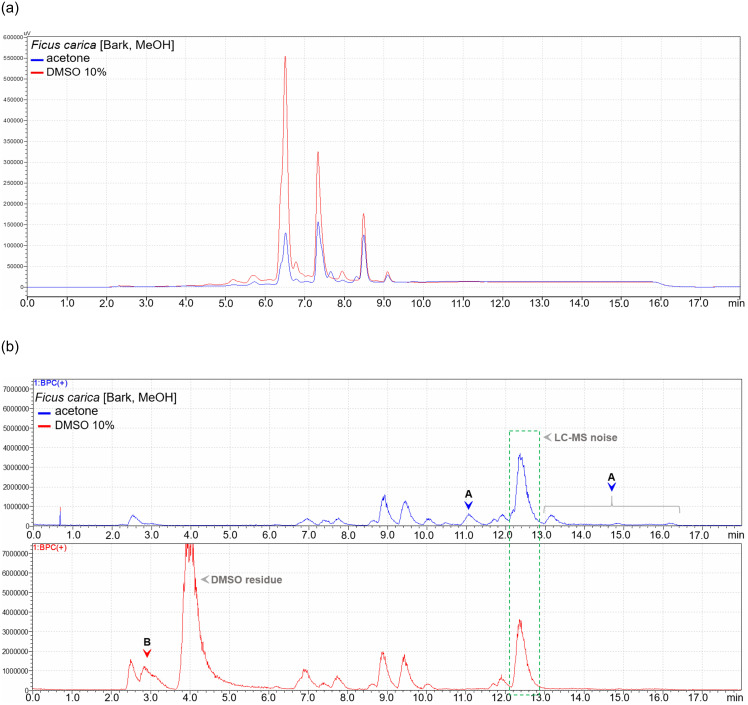
**a**. An overlay of UV chromatograms of acetone (blue) and DMSO (red) based working solutions of a methanol barks extract of *F*. *carica*, which had a better solubility in 10% DMSO. Higher quantities of phytochemicals are seen in the in a DMSO based working solution. **b**. Comparison of base peak chromatograms of acetone (blue) and DMSO (red) based working solutions of a methanol barks extract of *F*. *carica*, which had a better solubility in 10% DMSO. At least 4 (marked A) and 1 (marked B) additional compound(s) are visible in the chromatograms of acetone and DMSO based working solutions, respectively.

Furthermore, Fig 3a exemplifies the observation of higher quantities of phytochemicals in a DMSO dissolved working solution of the *F*. *carica* bark extract with higher solubility in 10% DMSO. The differences are particularly higher towards the more polar region of the chromatogram. The BPC of the same pair of working solutions showed 4 additional compounds as compared to 1 in acetone and DMSO based working solutions, respectively (Fig 3b). Noteworthy is also the lesser polarity of additional compounds in acetone as compared to DMSO based working solutions.

### Accounting for aspects likely to affect reproducibility

**Fig 4:**
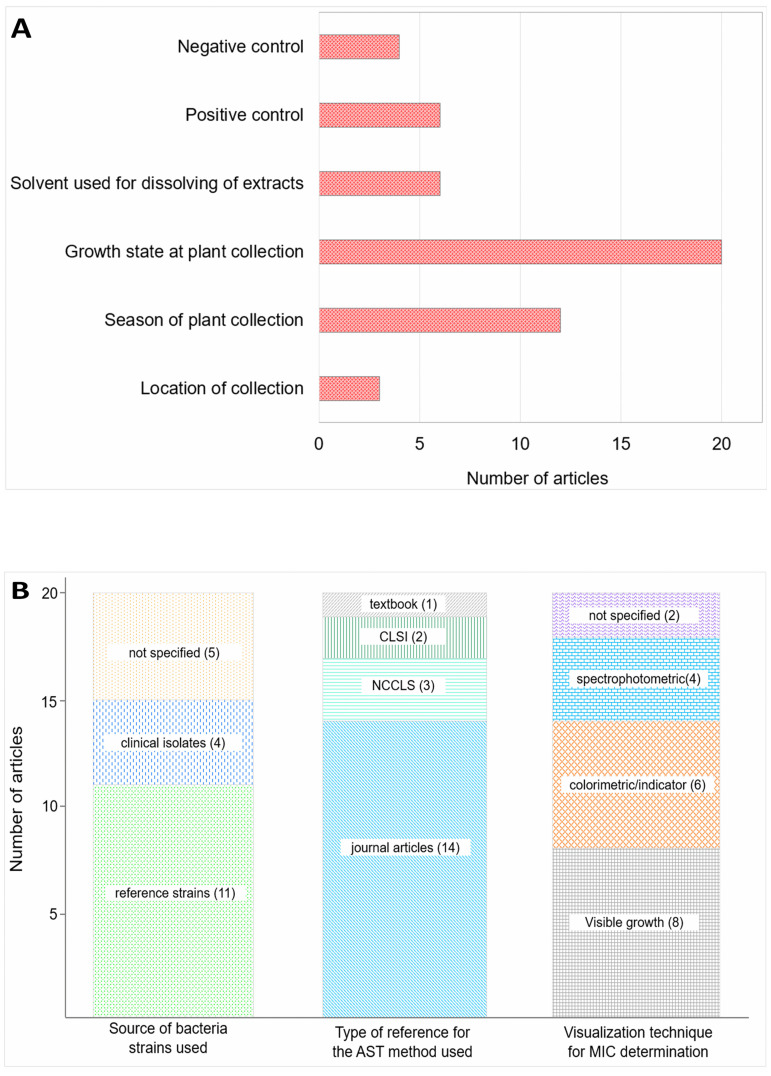
**a**. Frequency of missing key information/aspects in the referred articles (n = 20). **b**. Variations in methodological approaches in antimicrobial susceptibility testing with respect to identities of studied bacteria, origins of test methods and visualization techniques for ascertaining the MIC values (n = 20).

Upon evaluation of selected aspects in the articles used to determine our choices of the studied plants, several inconsistencies were observed:

1. On aspects related to practices during the collection of plant materials; 13 out of 20 articles did not specify the time of the year/season in which the collection was done. Additionally, the growth state of the plant at the time of collection (e.g. maturity, flowering) was not indicated in all articles. On the other hand, 18 out of 20 articles adhered well to the reporting of the location(s) from which the studied plant(s) were collected (Fig 4a).
2. Moreover, 11 out of 20 articles indicated to have used reference bacterial strains from sources such as the American Type Culture Collection (ATCC), Microbial Type Culture Collection (MTCC), National Collection of Industrial Microorganisms (NCIM), Center of Institut Pasteur (CIP) or PMFKg. Also, 5 out of 20 articles reported the use of only clinical isolates. Further, 4 out of 20 articles did not specify the sources of the bacteria used in conducting the AST studies (Fig 4b).
3. Regarding methodological aspects in the conduction of antimicrobial susceptibility tests; 6 out of 20 articles did not specify the solvent(s) used in dissolving the crude extracts before testing. Only 5 out of 20 articles cited the Clinical and Laboratory Standards Institute (CLSI), formerly called the National Committee for Clinical Laboratory Standards (NCCLS). The remaining articles (16 out of 20) indicated to have cited other journal articles or a textbook as a reference for the applied test methods (Fig 4b).
4. Furthermore, we noted varying methods used in evaluating the MIC values of the studied extracts. These included unaided visual observation for turbidity, colored indicator-aided visual observation, and the use of spectrophotometric devices. Additionally, the positive and/or negative control(s) used in the AST experiments were missing in 8 out of 20 evaluated articles (Fig 4b).

## Discussion

### Antibacterial activity of identical plant species

Low chances of reproducing previously reported antibacterial activities can be anticipated even upon ensuring the use of the same plant, bacteria species, extraction solvents, studied plant parts, and testing methods. We could not reproduce any of the previously reported MICs of the tested plants against the tested bacteria. The MICs we observed in *Cinnamomum verum* L. and *Piper betle* L. were at least four folds higher than those previously reported (Table 2). This was irrespective of ensuring the use of same plant species and extracting solvents, using recommended solvents for dissolving extracts, and employing reference bacterial strains with no known resistance mechanisms. With some variations in the extraction and MIC reading methods, a small degree of variation in the MIC values was expected. However, our results showed relatively high deviations from those previously reported (Table 2).

On the one hand, reproducibility challenges are due to solubility issues of crude extracts and varying composition of phytochemicals due to differences in geographical locations, sampling, climatic conditions, and ecological factors [9, 10, 42, 43]. On the other hand, this might be due to unstated plant-related and experimental details such as antibacterial testing methods and used bacterial strains [13, 14]. To account for climatic conditions, plants used in this study were grown in conditions simulating their usual environments in terms of temperature and relative humidity (Table 1). Other challenges will be discussed in more details.

### Solubility of plant extracts

Crude extracts from extracting solvents of varying polarities dissolve better in acetone as compared to the commonly used DMSO 10% solution. For instance, 17 out of 24 extracts showed better solubilities in acetone during the preparation of stock solutions (Table 4). However, the MIC values of extracts dissolved in acetone were generally not different from those dissolved in DMSO (Tables 2 and 3).

Since most of antibacterial plant-derived compounds are of low to intermediate polarities [14, 44–46], acetone is well suited for uses in extracting and dissolving of crude extracts from plant matrices. This is as well due to its good miscibility with water and non-toxicity to bacteria at higher concentrations (25% v/v) [20, 21, 47]. On the other hand, the use of DMSO offers better compounds’ stability in solution, as well as lower vapor pressure. These features are crucial when prolonged storage or testing times are needed [48].

Insufficient solubility of the many extracts in 10% DMSO prompted our undertaking to use acetone in order to avoid missing out compounds with antibacterial activity. LC-MS analysis showed qualitatively similar compositions of the working solutions prepared from the two solvents. Further, a better solubility of an extract in a particular solvent was related to the presence of higher quantities of corresponding phytochemicals in the resulting working solution (Figs 1–3b). However, some extracts indicated additional compounds in acetone dissolved extracts’ solutions, and most likely did not miss any compounds present in 10% DMSO dissolved solutions.

Since the solvent used for extraction as well as the diluent have an influence on the composition of the resulting test/working solution, a careful selection of the two is necessary. Based on quantitative benefits, our findings are suggestive of favoring acetone as a diluent when handling extracts obtained from less polar solvents and using 10% DMSO for extracts from more polar organic solvents.

### Plant materials, sampling and phytochemicals composition

Reporting of essential details about plants used in the screening for antibacterial activities is inadequate in a big proportion of published research articles. This is demonstrated by the missing information on the season and location of collection, as well as the maturity state of plants at collection in a big number of referred studies (Fig 4a). This challenge is aggravated by the observed low reporting of both qualitative and quantitative phytochemical profiles of the studied extracts. Since plants contain different types and quantities of phytochemicals in different seasons and at different maturity stages, stating of these details is crucial [9, 11, 42, 43]. The lack of this information results in less objective plant collection, and contributes to low reproducibility.

However, the amount and types of phytochemicals in plants are likely to vary even during different hours of a day [49]. The availability of details of season and plant’s stage of maturity at collection may therefore not address this challenge in full. Factors such as the amount of sunlight and water received, soil type, and predators or pathogens induction of phytochemicals production may also largely vary in different geographical locations even during a similar season [9–12]. This is examplified by the negative tests for sterols and caffeic acid in methanol leaves extract of *V*. *album* and acetone leaves extract of *O*. *europaea* respectively, as opposed to the corresponding previous reports. The absence of sterols in the *V*. *album* extract may have contributed to the observed discrepancies in antibacterial activities. However, this cannot be stated with certainty because the initial study did associate the observed antibacterial activities to any of the phytochemicals found in the extract. On the other hand, caffeic acid was shown to have inhibitory activity againt *E*. *coli* and *K*. *pneumoniae* among other bacteria. The differences in antibacterial activities observed in *O*. *europaea* leaves extracts can therefore be related to the missing sole or synergistic role of caffeic acid [22, 24].

Taken together, it is of great necessity to indicate the season and state of maturity alongside location(s) of plant collection. This will enable others to make all possible adjustments towards conforming to the previously reported conditions. Moreover, doing qualitative and/or quantitative fingerprint profiling of tested extracts using techniques like Thin Layer Chromatography (TLC), High Performance Liquid Chromatography (HPLC), or Liquid Chromatography-Mass Spectrometry (LC-MS) among others, should be considered necessary. Using fingerprint profiles enables an objective comparison on the extents at which the extracts to be studied are similar to those used previously.

### Testing for antibacterial activities

There is a limited use of reference bacteria isolates in carrying out antimicrobial susceptibility testing of crude extracts from plants. Moreover, uncomprehensive reporting of methodological aspects and the use of varying references for methods of antimicrobial susceptibility testing are common.

Our evaluation revealed that 9 out of 20 referred articles either indicated the use of clinical isolates or did not give any reference(s) of the studied bacteria (Fig 4b). The use of clinical isolates largely limits the reproducibility of obtained results by researchers elsewhere. Even upon successfully reproducing other factors, the genetic and phenotypic variations among clinical isolates of a particular bacterium may hinder the objective screening of plant extracts for antibacterial activity [14]. Lower susceptibilities to plant extracts have been observed in tests that involved the use of clinical isolates of bacteria or those with known resistance phenotypes [50–52].

Furthermore, we observed missing details on the solvent(s) used in the preparation of extracts’ test solutions as well as on the applied positive and/or negative controls used (Fig 4a). The non-disclosure of solvents used in preparing test samples disguises a proper choice of solvent(s) in the follow-up studies. Moreover, the use of solvents toxic to bacteria such as methanol and ethanol, or using recommended solvents above optimal concentrations precipitates the reporting of false-positive results [13, 14, 53]. The lack of information on the used negative controls aggravates the magnitude of this challenge.

Additionally, the approaches in the testing methods may be widely varying due to the observed diversity of references of methods for antimicrobial susceptibility testing. Among the 20 articles we referred to, 16 cited other published research papers as a reference for the used AST method (Fig 4b). Since each cited article might have done slight modifications of a previously reported method(s), this is also likely to affect the reproducibility [13, 14].

To warrant good reproducibility, it is therefore necessary to ensure the use of reference bacterial strains, especially during initial screening of plant extracts for antibacterial activities. This will enable others to select objectively the type of bacterial strains to use during the follow-up studies. Additionally, a thorough reporting of key aspects of the AST experiments and the use of standard methods from bodies like CLSI and EUCAST is a commendable approach in safeguarding the reproducibility of reported results [13, 18, 19].

### Antibacterial activity of related plant species

Most interestingly, we found more extracts with higher antibacterial activity in the species related to those primarily identified in our literature review. This indicates that, screening of related species for antibacterial activities is a good approach in the search for antibacterial hit compounds from plants. As shown in Table 3, antibacterial activities were observed in the extracts of *G*. *spicata and P*. *officinalis*. While the MIC values exhibited by *G*. *spicata* extracts were at least 6.5 higher than those previously reported in *G*. *smeathmannii*; that of *P*. *officinalis* extract against *K*. *pneumoniae* (128 μg/mL) was about two times lower than that previously reported in *P*. *broteroi* (250 μg/mL). Additionally, the antibacterial activity of the acetone extract of *P*. *officinalis* against *E*. *coli* (MIC = 256 μg/mL), was not seen previously.

Expanding the search perimeter by screening of species related to those with previously reported activities is therefore noteworthy [14, 54]. This approach is generally better than the common practice of selecting plants based on their traditional uses in the treatment of bacterial infections. This is underscored by the reports that antibacterial activities observed among randomly collected plants did not significantly differ from those being collected on the bases of their usage by traditional healers [14].

## Conclusion

The global rise of antimicrobial resistance demands for diversified approaches in the search for novel antibacterial agents. Plants, among other natural sources, host a great potential in contributing to the discovery of new antibiotics. As it might be common among other research groups, we have observed a very low reproducibility of previous findings on antibacterial activities of plant extracts. We also noted inconsistencies and a wide variation in the amount of provided information regarding experimental procedures, plants, and bacterial strains used. Although poor reproducibility depreciates the usefulness of the initial efforts and discourages follow up works, plants remain to be a potential source of novel antibacterial agents. This necessitates putting in place adequate and collective measures in facing the reproducibility challenge.
